# Elemental Speciation Analysis in Environmental Studies: Latest Trends and Ecological Impact

**DOI:** 10.3390/ijerph182212135

**Published:** 2021-11-19

**Authors:** Mauricio Llaver, Emiliano F. Fiorentini, María N. Oviedo, Pamela Y. Quintas, Rodolfo G. Wuilloud

**Affiliations:** Laboratorio de Química Analítica para Investigación y Desarrollo (QUIANID), Facultad de Ciencias Exactas y Naturales, Instituto Interdisciplinario de Ciencias Básicas (ICB), Universidad Nacional de Cuyo, CONICET/UNCUYO, Padre J. Contreras 1300, Mendoza 5500, Argentina; mauri.llaver@gmail.com (M.L.); emifranfiorentini@gmail.com (E.F.F.); natyoviedo3189@gmail.com (M.N.O.); pamequintas@gmail.com (P.Y.Q.)

**Keywords:** sample preparation, green chemistry, chromatography, atomic spectrometry, electrochemistry, environmental samples, analytical methods, legislation

## Abstract

Speciation analysis is a key aspect of modern analytical chemistry, as the toxicity, environmental mobility, and bioavailability of elemental analytes are known to depend strongly on an element’s chemical species. Henceforth, great efforts have been made in recent years to develop methods that allow not only the determination of elements as a whole, but also each of its separate species. Environmental analytical chemistry has not ignored this trend, and this review aims to summarize the latest methods and techniques developed with this purpose. From the perspective of each relevant element and highlighting the importance of their speciation analysis, different sample treatment methods are introduced and described, with the spotlight on the use of modern nanomaterials and novel solvents in solid phase and liquid-liquid microextractions. In addition, an in-depth discussion of instrumental techniques aimed both at the separation and quantification of metal and metalloid species is presented, ranging from chromatographic separations to electro-chemical speciation analysis. Special emphasis is made throughout this work on the greenness of these developments, considering their alignment with the precepts of the Green Chemistry concept and critically reviewing their environmental impact.

## 1. Introduction

Life sciences constantly profit from innovative analytical methods. To this point, these methods mainly refer to the identification, separation, and quantification of chemical entities in samples as diverse as water, soils or sediments, plants, biological fluids, tissues and organs, among many others [1]. In this context, the concept of speciation analysis has become extremely popular lately, usually being applied to studies related to the distribution of a chemical element in its different forms or species. The International Union of Pure and Applied Chemistry (IUPAC) has taken on the task of providing an unequivocal definition for this term, describing it as the “analytical activities of identifying and (or) measuring the quantities of one or more chemical species in a sample” [2]. It is precisely in relation to this that the development of highly selective and sensitive analytical methods for the identification and determination of toxic and/or essential elemental species constitutes one of the main demands on modern analytical chemistry [3].

The concept of speciation has become extremely important in environmental studies, as it provides information related to the bioavailability and toxicity of a given element and has demonstrated that these factors depend not only on the nature of the element itself and its concentration, but also on its species and chemical combinations present in the systems under study. For example, organomercury compounds, such as methylmercury (CH_3_Hg^+^), present a much greater toxicity than inorganic forms of mercury (Hg^2+^), and there are even cases in which a certain species is considered a micronutrient (Cr^3+^), whereas another one is classified as carcinogenic (Cr^6+^) [4,5]. It is therefore evident that the speciation analysis of an element present in an environmental sample is a fundamental need in order to obtain exact results on the risk of exposure, especially considering that these species can be highly toxic and can enter food chains through different pathways [6].

Taking this into account, the ongoing development and application of advanced analytical methods are crucial activities in current analytical laboratories to identify and quantify species in concentrations in the order of traces (µg L^−1^ or lower) [7]. This is precisely one of the main challenges currently faced by analytical scientists, due to the low concentrations in which these elements are found in nature, added to the fact that each species represents only a fraction of the total; thus, highly sensitive and precise methods to obtain trustworthy results are needed. In this sense, the combination of sample treatment (e.g., liquid-liquid, solid phase microextractions), highly effective separation systems (e.g., liquid or gas chromatography), and sensitive elemental detection techniques (e.g., atomic spectrometry, inductively coupled plasma-mass spectrometry) represents the most valuable tool to face this task.

This review aims to summarize the latest developments in the field of metal and metalloid speciation analysis in environmental (water, soil, and sediment) matrices, with the spotlight set on reports from the last five years. In this sense, although air represents a very interesting environmental matrix as well, there are no speciation analysis reports involving this type of samples for the hereby presented analytes in the evaluated period. The discussion is centered around each individual element but includes descriptions and in-depth discussions on the latest methods and techniques, including sample preparation approaches, instrumental separations, elemental detection, and electroanalytical developments. Special emphasis is made throughout this work to critically evaluate the greenness of the presented methods and to assess their environmental impact, making it not only a descriptive review of analytical applications, but also an integrated analysis of their novelty and sustainability.

## 2. Legislation

**Figure 1:**
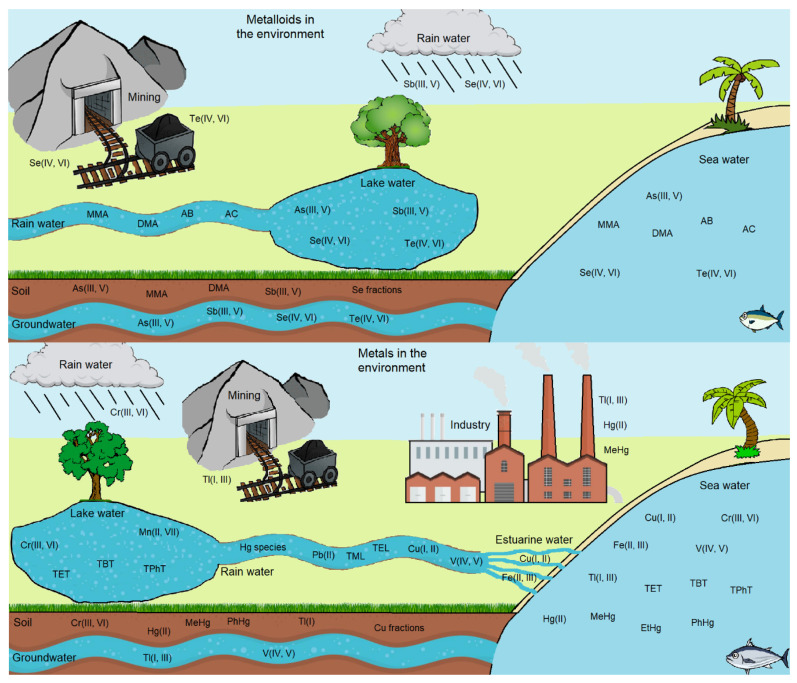
Metal and metalloid species distribution in diverse environmental and industrial compartments. AB: arsenobetaine, AC: arsenocholine, DMA: dimethylarsinic acid, EtHg: ethylmercury, MeHg: methylmercury, MMA: monomethylarsonic acid, PhHg: phenylmercury, TBT: tributyltin, TEL: tetraethyl lead, TET: triethyltin, TML: tetramethyl lead, TPhT: triphenyltin.

The rapid advancements of industry and technology are, without a doubt, a defining aspect of modern-day societies. However, although their influence on our day to day is ever present, their impact on the environment is usually ignored or understated by the general public. Even though natural sources of chemical, physical, and pathogenic polluting agents exist (e.g., weathering, plant and animal decomposition, volcanoes), anthropogenic influence has a much more significant impact on water, soil, and air contamination [8]. Among these, the contamination of water and soil with metals and metalloids has become one of the most serious modern environmental problems, due of the toxic nature of many of them even at trace levels [9]. Figure 1 shows a schematic representation of the distribution of the metal and metalloid species analyzed in this review, in terms of their distribution in different environmental and industrial compartments.

The toxicity of several metal and metalloid species has led to more and more stringent regulations from governments in an attempt to limit the impact of human activities on the environment, including some advances in recent years towards a species-guided legislation. This has been a direct consequence of improvements in toxicological studies, both in terms of extent and reliability, which have provided the tools for legislators to set maximum acceptable limits for certain relevant species, instead of for total elements or, as was usually expressed, “an element and its species” [10]. One of the earliest cases was the individual regulation of inorganic Cr species, which are legislated separately by virtually all regulatory agencies since the decade of the 1990s, recognizing the significantly higher health risks associated with Cr(VI) over Cr(III). As examples, the United States Environmental Protection Agency (U.S. EPA) established in 1995 recommended maximum concentrations in freshwater for Cr(VI) and Cr(III) of 16 and 570 µg L^−1^, respectively, whereas Australia and New Zealand establish limits only for Cr(VI) in freshwater (in a range between 0.01 and 40 µg L^−1^, depending on the local level of protection) and for both species in saltwater [11]. These species are also usually regulated in soils. For example, the Canadian Council of Ministers of the Environment (CCME) has differentiated between Cr(VI) and total Cr in agricultural, residential, commercial, and industrial soils since 1999, allowing maximum concentrations of 1.4 mg kg^−1^ (dry weight) for Cr(VI) and 87 mg kg^−1^ (dry weight) for total Cr [12].

More recently, speciation-based environmental legislation was developed for other elements, such as Hg. This element is triply regulated in soils by the UK’s Environment Agency, which distinguishes between elemental Hg, inorganic Hg(II), and CH_3_Hg^+^, setting guideline values of 1.0, 170, and 11 mg kg^−1^ for these in residential soils, reflecting on the one hand the higher toxicity of methylmercury over inorganic Hg(II) and, on the other, the dangers of the vapor inhalation pathway of elemental Hg [13]. Tin (Sn) is another metal that is differentially regulated, the most usual distinction being that between inorganic Sn and tributyltin (TBT). The latter was completely banned in 2013, after over 40 years of use as a biocidal in anti-fouling paints for the hulls of oceanic vessels, from which it slowly leaks into the water, where it is highly toxic for several organisms [14]. This is why species-specific legislation was put in place concerning TBT, limiting its concentration to values such as the European Union’s (EU) maximum allowable environmental quality standard in environmental waters of 1.5 ng L^−1^ and the U.S. EPA recommendation of 72 ng L^−1^ for freshwater and 7.4 ng L^−1^ for saltwater [15,16]. Meanwhile, the CCME establishes freshwater limits for both tributyltin (8 ng L^−1^) and triphenyltin (22 ng L^−1^), another organotin compound [17].

Nevertheless, speciation is not considered in environmental legislation concerning other elements that are relevant to human and animal health, such as arsenic, which is regulated in terms of total elemental concentration all over the world. In this sense, Feldmann and Krupp suggested a potential strategy for routine analysis and legislation in 2011, differentiating three fractions: toxic inorganic As, non-toxic arsenobetaine, and the potentially toxic fraction that includes all other organoarsenicals [18]. This strategy, although erring on the side of caution due to the fact that not all organoarsenicals are considered toxic, was recently questioned by one of the original authors in light of new toxicological data on some lipid-soluble As compounds that demonstrates that, although useful, species-driven legislation is not necessarily simple to establish [19]. Similarly, selenium (Se) is an element that is legislated in terms of its total concentration, although it is known that its characteristics, including its toxicity, strongly depend on the species it is present as [20]. As is the case with As, the complexity of the environmental Se toxicity pathways is the main reason that hinders the development of a species-focused legislation. This generates inconsistencies, due for example to the major bioconcentration occurring between the water and the lowest trophic level (usually algae), which depends strongly both on the species of algae and the species distribution of Se [21]. The extent of these inconsistencies is such that the U.S. EPA modified its guidelines recently, considering fish egg/ovary Se as the primary indicator when examining a body of water, rather than looking at the water concentration alone [22]. This proves, once again, the difficulties associated to the development of a legislation based on species.

It is also relevant to point out that some elements that have only recently found large-scale applications are still not directly legislated in terms of their presence in the environment. Such is the case of tellurium, for which there are indirect regulations concerning the recovery and recycling of photovoltaic panels (by far the main use for this element), but not any established threshold levels for its presence in water or soils, let alone of its species [23].

## 3. Remarks on the Greenness of Speciation Analysis Methods

As was noted, speciation analysis requires the development of highly selective and sensitive analytical methods, which have been performed mainly by coupling chromatographic techniques and sensitive element-specific detectors. Additionally, non-chromatographic techniques have also been developed, albeit with a more limited separation power, usually restricted to specific species. Both approaches require the continuous development of improved methods, which must consider their environmental impact as a key parameter to optimize, especially because their main concern is precisely the study of the environment. In this sense, the guidelines of Green Chemistry represent an interesting standpoint from which to evaluate the potential effects of the application of developed methods and/or techniques [24].

At present, research on Green Analytical Chemistry is increasing significantly, however, there is little critical reflection in these studies about the environmental sustainability of the practices that involve elemental speciation analysis. To develop an environmentally friendly speciation analysis method, it is necessary to avoid the use of significant amounts of solvents and chemical reagents and to reduce the generation of waste. These requirements have not been satisfactorily met through the development of the so-called hyphenated analytical methods which, despite this detail, have hitherto occupied a central place in the field of speciation analysis, mainly due to their practicality, easy automation, and analytical frequency [25].

On the contrary, elemental speciation analysis based on non-chromatographic techniques has managed to better adapt to the concepts established by Green Chemistry. In this sense, the application of microextraction techniques and novel sorbents and solvents designed for miniaturized methods has minimized the use of reagents, which is beneficial for the environment and, additionally, has increased the analytical sensitivity through the preconcentration of the evaluated species. Furthermore, miniaturization has also allowed the use microvolumes of solvents, which can be completely injected into elemental detectors that decompose them, thus significantly reducing waste production, even in cases in which low-efficiency pneumatic nebulizers are used for sample introduction [26]. Finally, it is important to highlight that the use of greener alternative solvents for these microextraction techniques is becoming increasingly common, with currently widely known families such as ionic liquids (ILs) and deep eutectic solvents (DESs), as well as the use of more efficient nanosized solid sorbents that can be recycled [27].

**Figure 2:**
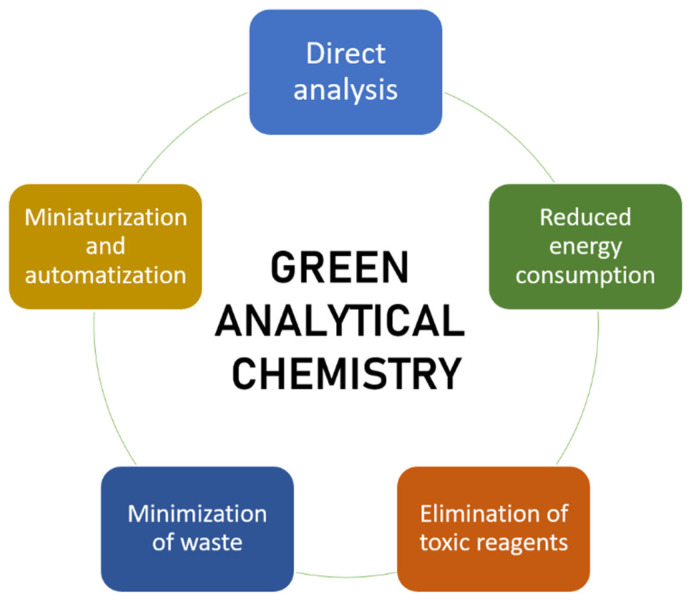
Summary of the most important aspects considered by the different scales when evaluating the greenness of analytical methods.

The choice between the proposed methodological approaches for speciation analysis depends on several factors, including the number of species to be evaluated and the required analytical sensitivity. However, the greenness of the methods should also be an important parameter to consider in this context. In order to evaluate this, different scales have been defined over the years, including the concept of Eco-Scale [28]. For this, the nature and dangers related to the reagents, the energy consumption, and the waste generation are evaluated. A modification of this eco-scale, denominated Green Certificate, adds a color code according to the degree of greenness of the studied approach. Finally, a new analytical tool has recently been proposed, called the Green Analytical Procedure Index, which evaluates the green character of the entire analytical process, from sample collection to final determination [29]. A summary of the considered parameters for determining these indexes is presented in Figure 2.

**Figure 3:**
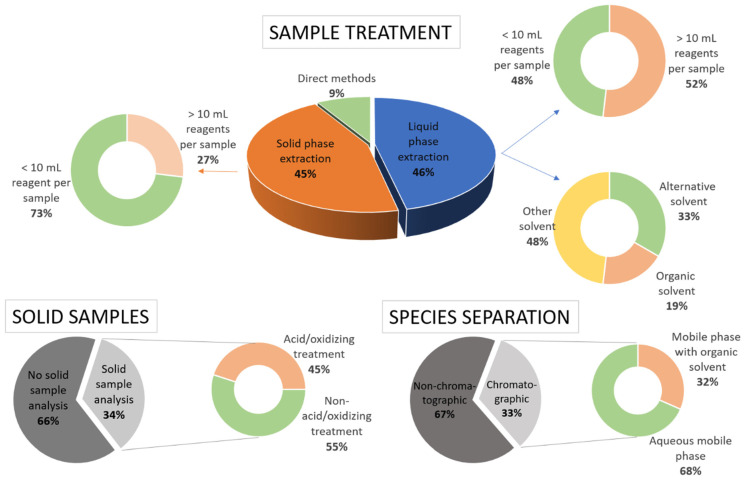
Distribution of diverse factors affecting the environmental impact of the reports included in this article.

Although these indexes are still not widely applied, especially by authors evaluating their own work, this review aims to highlight both the negative and positive aspects of the presented methods regarding their environmental impact. In this sense, parameters similar to those on which these indexes are based will be presented and analyzed, in an attempt to go one step further from the analytical aspects of the reviewed publications, and the increasingly relevant aspects of method greenness will be evaluated. Figure 3 shows a summary analysis of diverse factors related to the environmental impact of the work reviewed in this article. For the elaboration of this figure, the 10 mL of reagents threshold and the classification of alternative solvents (those different from classic organic solvents and/or strong acids or bases) have been taken from the analytical Eco-Scale concept proposed by Gałuszka et al. [28].

## 4. Metalloid Speciation Analysis in Environmental Samples

### 4.1. Antimony

Antimony (Sb) is a non-essential element widely distributed in environmental compartments, considered a contaminant of great concern by the U.S. EPA and the EU [30]. The acute toxicity of Sb involves gastrointestinal and respiratory symptoms, and prolonged intake of this element is associated with skin and cardiovascular diseases and lung cancer [31]. However, it was found that the toxicity and bioavailability of Sb depend strongly on its oxidation state and the nature of its species, as inorganic Sb species are more toxic than its organic compounds, whereas Sb(III) is more toxic than Sb(V). Consequently, its speciation analysis is highly necessary to investigate the risk and the environmental impact related to its occurrence. In this sense, given the fact that Sb is usually present at or below trace concentrations in environmental samples, preconcentration methods are necessary prior to its determination by an instrumental technique [32].

Different strategies for Sb speciation analysis have been reported in recent years, as can be seen in the summary presented in Table 1. One of them involved the use of hybrid nanoparticles (NPs) composed of oxidized multi-walled carbon nanotubes (MWCNTs) and nano-TiO_2_ as solid sorbent for the flow injection analysis of river water, coupled to electrothermal atomic absorption spectrometry (ETAAS) detection [33]. This method required low sample and reagent volumes compared to other SPE-based approaches, although several concentrated strong acids were used for the synthesis of the sorbent, producing a considerable amount of acidic waste. In another case, the preconcentration and determination of Sb species was performed with ammonium pyrrolidinedithiocarbamate (APDC) as complexing agent and the non-ionic surfactant Triton X-114 as extractant by cloud point extraction (CPE) with ETAAS detection in river water samples. The authors highlighted the use of the surfactant as an eco-friendly solvent for the preconcentration and selective extraction of Sb(III). For the speciation analysis, total Sb was determined after using L-cysteine to reduce Sb(V) to Sb(III), and the concentration of Sb(VI) was calculated by difference [32]. In another work, diethyl dithiophosphate (DDTP) was used as a specific chelating reagent for Sb(III), and a magnetic IL (MIL) was applied for its extraction, followed by ETAAS [34]. In this work, KI was used for the selective reduction of Sb(V) to Sb(III) and the use of the MIL avoided the need for flammable and volatile organic solvents commonly used in liquid-liquid microextractions (LLMEs). Additionally, the optimization of the method by a multivariate study decreased the number of necessary experiments and, consequently, the reagent consumption and waste production.

Additionally, Sb is a highly volatile element usually associated with atmospheric particulate matter (PM), making its speciation analysis in these samples highly important regarding its effects on human health. A method for the determination of Sb(III) and Sb(V) in inhalable PM2.5 by high-performance liquid chromatography coupled to inductively coupled plasma-mass spectrometry (HPLC-ICP-MS) has been developed [36]. In this work, a sequential extraction based on a reducing step with hydroxylammonium chloride, acetic acid, and EDTA followed by an oxidizing extraction with HNO_3_ and EDTA was applied. The results showed recovery values from 90.0 to 110.0%, although a considerable volume of concentrated HNO_3_ was used as extraction solvent, which could be associated with a significant environmental impact.

### 4.2. Arsenic

Arsenic is classified as a metalloid with a significant environmental presence, a component of hundreds of minerals. Its compounds are used in various industrial applications and as pesticides and herbicides, among other applications. Arsenic exposure can affect various organ systems to the point of death, and the element is considered a human carcinogen, characteristics that have made it a worldwide concern for human health and the environment. It is found in the oxidation states −3, 0, +3, and +5, forming part of inorganic and organic compounds, with inorganic forms being more toxic than organic ones and, among the former, As(III) compounds are more toxic than As(V) ones [57]. The different toxicities and distributions of As species in the environment makes their individual quantification necessary, rendering the analysis of total As alone inadequate. However, this represents a complex task, due to the significant number of species to identify, their similar chemical and physical properties and their low concentrations, making the use of sensitive methods and techniques necessary [58].

Among these techniques, it is worth highlighting an interesting approach based on the DES choline chloride-phenol, an environmentally friendly solvent, for the microextraction of As(III) and As(V) by means of ultrasound-assisted LLME in samples of lake and river water, sediment, and soil. Diethyldithiocarbamate (DDTC) was used as a complexing agent for As(III), whereas As(V) required a reduction step prior to its complexation as part of the speciation analysis strategy [37]. Furthermore, the use of ILs and, more specifically, MILs, has also been exploited for As speciation analysis. For example, an air-assisted LLME method based on the MIL [C_4_mim][FeCl_4_] was used for the highly selective separation of traces of As(III) and As(V) in pond and river water, sediment and soil. In this case, As(III) was complexed with APDC, whereas As(V) was again subjected to a previous reduction step for determination by difference. This approach avoided the use of an organic solvent as a dispersant by using air to promote an efficient contact between the MIL and the sample solution but, however, the soil and sediment samples required strong acids and oxidants for their digestion [38]. In addition, a dispersive LLME (DLLME) method using MILs was recently developed for the speciation analysis of inorganic As in dam, river, sea and underground water, sediment and soil. This approach also involved APDC as a complexing agent for species separation; however, the extraction was carried out in a strongly acidic medium and required the dilution of the MIL with chloroform prior to its injection into the ETAAS detector. No acid digestion of the samples was required in this case and, instead, a clean-up procedure that used a minimal amount of chloroform was proposed [39].

In these three LLME-ETAAS approaches based on alternatives solvents, it was possible to determine inorganic As species in concentrations lower than the maximum permissible limits established by international recommendations. The results pointed to a predominance of As(V) with respect to As(III), suggesting the need of continuous monitoring to avoid deterioration with possible future increases in the content of these species.

Based on the important number of organic As species, chromatographic techniques such as HPLC are most adequate in some cases, usually coupled to ICP-MS, which presents high ionization efficiencies, low matrix interferences, high selectivity and wide dynamic ranges for sensitive As detection [59]. One of these was based on a simple and fast DLLME method with octanol as a low-density solvent for the extraction of As(III), As(V), and phenylarsenates in lake and pond water samples. For this analysis, significant amounts of extraction and dispersion solvents were used, as well as large volumes of methanol in the chromatographic mobile phases. In addition, a digestion procedure that required 6 mL of concentrated HNO_3_ per sample was required for the determination of total As [40]. An interesting comparative work has also been published, highlighting the differences in the distribution, partition, and bioaccumulation of As species in fresh and salt water systems and their sediments. This work was based on the analysis of As(III), As(V), and the organic species monomethylarsonic acid (MMA), dimethylarsinic acid (DMA), arsenobetaine (AB) and arsenocholine (AC) without prior extraction or preconcentration. It should be noted that the determination of total As required only dilute HNO_3_ for the digestion of the samples and that organic solvents were not used in the proposed mobile phases, making this a method well adapted to the guidelines of Green Chemistry [41]. Finally, an analysis of the extraction and speciation of roxarsone (a food additive used in the animal production industry) and its metabolites was recently developed and applied to soil samples. These required a previous treatment with a mixture of strong acids, whereas the used mobile phase contained a low percentage of methanol [42]. In all of these HPLC-ICP-MS studies, a predominance of the inorganic species of As was found, although the concentrations were in all cases below the legislated limits regarding the total allowable content of As.

Finally, the combination of supercritical fluid extraction (SFE) and GC has also been proved to be useful for the speciation analysis of As [43]. In this report, the authors performed the extraction of DMA, MMA, and inorganic As from soils and sediments using supercritical CO_2_ with methanol as modifier and a microemulsion containing Triton X-405, cyclohexane, and butanol. Additionally, thioglycolic acid *n*-butyl ester was added to the extraction mixture for the in situ derivatization of the studied species, allowing for their direct posterior analysis by GC. Although SFE is usually considered a fairly clean method, the use of cyclohexane as part of the microemulsion and as solvent for the collection of the derivatized species (2 mL per determination) affected the overall greenness of the approach.

### 4.3. Selenium

Se is an element of dual nature in the environmental and biological sense, since both its deficiency and excess can be harmful [60]. Se has been recognized as a fundamental component for the action of several enzymes with antioxidant functions in diverse metabolic pathways and, additionally, it has been suggested that some organic Se species could possess health benefits by preventing some types of cancer [61]. Nevertheless, its excess intake is also associated with risks, including central nervous system affects [62]. It is also important to point out that the toxicity and bioavailability of Se depend strongly on the species it is present as, because inorganic species are approximately 40 times more toxic than organic species and, among the former, Se(IV) toxicity is 10-fold higher than Se(VI) toxicity, making its speciation analysis key for understanding its effect on environmental compartments [63]. In this context, biological analyses are more concerned with the organic species, requiring hyphenated techniques such as HPLC-ICP-MS, as the number of species to determine can be in the hundreds [64,65]. Chromatographic techniques are usually replaced by more simple and accessible methods in environmental analysis, as inorganic species analysis is the most relevant. Hence, the combination of microextraction techniques and atomic spectrometry detectors have set a clear trend in the last five years, giving place to limits of detection (LODs) and sensitivities comparable to those of more advanced techniques, such as ICP-MS, albeit at a much lower cost [66].

Among these, the development of a solid phase extraction (SPE) method using a graphene oxide-TiO_2_ nanosorbent was recently reported for the speciation analysis of inorganic Se in spring water. The material selectively retained Se(IV) within the pH range between 2.0 and 10, allowing its exclusive extraction and determination by ETAAS after elution with only 0.9 mL of 3.0 mol L^−1^ HNO_3_. The speciation analysis in this case was based on the pre-reduction of a second aliquot of the sample, followed by the subsequent calculation of the concentration of Se(VI) by difference [44]. This pre-reduction is a common practice in the inorganic speciation analysis of Se and other elements, and it usually requires substantial amounts of concentrated HCl, which represents an important source of acid waste. Besides this, the developed method did not involve the use of organic solvents or major amounts of dangerous substances and, although the synthesis of the graphene oxide-TiO_2_ sorbent did involve significant amounts of strong acid (H_2_SO_4_) and oxidants (Na_2_S_2_O_8_, H_2_O_2_ and KMnO_4_), the high reutilization capacity of the material (up to 95 sorption-desorption cycles per each 30 mg) certainly ameliorated the impact of its preparation [67]. Firouzabadi et al. also developed a SPE method, albeit based on the magnetic solid phase extraction (MSPE) technique, using magnetized MWCNTs functionalized with Bismuthiol II as sorbent for the analysis of river and sea water [45]. Here, the authors not only simplified the microextraction process thanks to the magnetic properties of the material, but they also restricted the use of acids, requiring concentrated HNO_3_ only during the synthesis of the sorbent (the amount is not specified) and 0.5 mL of 1.0 mol L^−1^ of the same acid for the elution of the analytes. Nevertheless, given that speciation analysis was based on the selectivity of Bismuthiol II towards the extraction of Se(IV), the pre-reduction strategy with concentrated HCl was also required in this case.

In the face of the significant consumption of reagents involved in the preparation of the aforementioned covalently bonded sorbents, physisorbed materials stand as an interesting alternative to reduce the impact of their obtention. A nanosilica-IL hybrid that was straightforwardly prepared by mixing the nanomaterial and the IL in water, involving no acids, oxidants, or organic solvents, was recently reported [46]. One milligram of this hybrid nanomaterial was applied in a dispersive micro-solid phase extraction (D-µ-SPE) method followed by ETAAS detection, requiring only 100 µL of a Triton X-114 solution in ethyl acetate as eluent and no other solvents, making its waste generation minimal. This method was applied for the speciation analysis of inorganic Se in river, sea, underground, and rain water with excellent results. Nevertheless, as the speciation analysis strategy was based on the use of the complexing agent APDC, which is selective towards the Se(IV) species, the analysis of a second, pre-reduced aliquot, was necessary. A DLLME method has also been reported, applying long-chain alcohols for the determination of Se(IV) and Se(VI) in river water with colorimetric detection [49]. The facts that no halogenated solvents were used, that the overall solvent consumption was minimal, and that the extraction was undertaken in the context of the technique of floating drop solidification were quoted as the main reasons for its low environmental impact, although the application of an acidic pre-reduction stage for speciation analysis represents a negative aspect with respect to this.

If the pre-reduction step is to be avoided, other strategies can be applied to achieve the desired speciation analysis. For instance, fractionation processes, in which successive extractions with different solvents and/or conditions are performed to obtain separated species or fractions, can represent an interesting alternative in this context. This was the case of a sequential extraction protocol that was applied for the separation of Se(IV), Se(VI), and selenocyanate in mining wastewater [48]. The method avoided the reduction of the sample and was, in turn, based on the derivatization and extraction of each species in three successive steps, resulting in extracts that were mixed and analyzed by gas chromatography with mass spectrometric detection (GC-MS). This approach gave place to excellent LODs and sensitivities for the three species in samples with high concentrations of sulfate, nitrate, and chloride, such as mining wastewater. However, the greenness of the method was severely affected by the use of chloroform, an organochlorine solvent of widely known toxicity, for the extractions.

Aside from microextraction approaches, an interesting alternative was recently presented by Ding and co-workers, based on the photochemical vapor generation of SeH_2_ and its detection by UV-Vis spectroscopy after adsorption on Au nanoparticles [50]. In this case, the direct photovolatilization by addition of formic acid and UV irradiation allowed for the generation of SeH_2_ from Se(IV) exclusively, whereas the addition of nano-TiO_2_ allowed for the generation of the hydride from both Se(IV) and Se(VI), eliminating the need of any chemical pre-reduction stages. It is also noteworthy that Se concentrations as low as 0.08 ng mL^−1^ induced a color change that could be directly observed with the naked eye, and that the method had an acceptable performance regarding interferences, as demonstrated by its application to lake, river, and sea water.

Regarding soil analysis, the most usual approach is the application of fractionation processes. This results initially in a quantification in terms of groupings (e.g., water-soluble Se, ligand-exchangeable Se, organic matter-associated Se, elemental Se), but not in terms of individual species [51]. These protocols usually demand some steps with dilute acids or bases and a final step for total analysis that requires concentrated acids, such as HNO_3_ and/or HF, representing the least environmentally friendly stage [52]. Furthermore, these methods can be combined with techniques that probe electronic environments, such as X-ray absorption near edge structure, which can provide actual species distribution information in each fraction, albeit not without difficulties and imprecision, as the required fitting sensitivities cannot be attained when one species is predominant over the others [51].

### 4.4. Tellurium

Tellurium (Te) is one of the rarest elements in the earth’s crust, having been considered as a mere chemical curiosity until recently [68]. Nevertheless, it has nowadays become an essential component in diverse technological applications, such as solar cells, being labeled by the EU as the element with the highest expected demand in the 2020–2030 period from a group of 14 metals considered vital for the development of six strategic industries with low environmental impact [69,70]. As a consequence, the uses and demand of Te are expected to increase significantly in the near feature, making the analysis of its presence in the environment necessary, especially when considering that Te inorganic species are highly toxic and teratogenic. In this context, Te(IV) is significantly more dangerous than Te(VI), making their individual analysis the most relevant, as the presence of organic species is not expected other than in specific biological systems [71,72].

The extremely low concentration of Te in environmental samples has made the application of preconcentration methods a usual approach for its quantification. Such is the case of an IL-assisted CPE method developed for the preconcentration of Te(IV) and Te(VI) from diverse environmental water, soils, and sediments, with ETAAS detection [53]. In this work, the speciation analysis strategy was based on the selectivity of APDC as chelant for the Te(IV) species, and the extraction was performed with Triton X-114 and 1-octyl-3-methylimidazolium chloride mixed micelles. The authors highlighted the greenness of the method, based on the use of a non-toxic surfactant and of a minimal amount of IL for sample treatment, although the need for a pre-reduction step involving a considerable amount of concentrated HCl certainly added a negative point in this respect. The same authors also developed a MSPE method coupled to hydride generation atomic fluorescence spectrometry (HG-AFS) to achieve the speciation analysis of Te in water, soils, and sediments [54]. In this case, a magnetic nanocomposite based on a polymeric IL was used as sorbent and the separation of the species was based on the different acid-base behaviors of Te(IV) and Te(VI). The sample treatment method required only 500 µL of 5.0 mol L^−1^ HNO_3_, which generated no acidic waste, as it was subsequently reacted during the hydride generation step, whereas the synthesis of the nanocomposite was carried out in aqueous media, avoiding the use of volatile solvents or other hazardous substances. Nevertheless, the speciation analysis was once again achieved by means of a chemical reduction of a second aliquot, as has been previously discussed.

An interesting modification on the target analytes for Te speciation analysis was presented by García-Figueroa and collaborators, who achieved the quantification of Te(IV) and of CdTe NPs in superficial, lake, and ground water [55]. This method was based on the headspace single drop microextraction of TeH_2_, which was generated by reaction of Te(IV) with NaBH_4_ on an acidic aqueous solution of Au(III). The drop was then injected into a graphite furnace for ETAAS quantification. The speciation strategy involved the analysis of a second aliquot with the addition of iodide during the hydride generation step, which allowed the formation of TeH_2_ from both free Te(IV) and from the CdTe NPs. The authors highlighted that, although the method did not result in a LOD that could cope with the extremely low levels of Te in environmental samples, it represented a suitable and sensitive alternative for the fast speciation analysis of Te(IV) and of CdTe NPs, with minimal environmental impact.

A compelling work also studied the presence of Te species in soils from abandoned mines, which represent one of the most significant samples regarding the contamination with this element. In this case, the quantification was performed directly by a combination of micro focused X-ray fluorescence, X-ray diffraction, and X-ray absorption fine structure. This approach was not only extremely clean, as it did not involve any kind of chemical sample treatment, but was also very efficient to detect Te(IV) and Te(VI) in the samples. The authors highlighted the fact that the element was unevenly distributed throughout the soil, being mostly present in “hotspots” associated to Fe hydroxides, with an average Te(VI):Te(IV) ratio of 2:1 [56].

## 5. Metal Speciation Analysis in Environmental Samples

### 5.1. Chromium

Chromium (Cr) is one of the most abundant metals in the Earth’s crust and it has diverse industrial applications. It can be found in nature under two stable oxidation states, Cr(III) and Cr(VI), which are a clear example of the different physiological properties that the species of an element can possess [73]. Cr(III) is essential for living organisms, as it enhances the activity of certain enzymes and stimulates the synthesis of cholesterol and fatty acids. In contrast, Cr(VI) is considered carcinogenic and mutagenic, and it is present in the environment as an industrial pollutant [74]. Thus, the opposing effects presented by Cr(III) and Cr(VI) require the development of analytical techniques that allow the quantification of both species in environmental samples for a correct evaluation of the degree of toxicity. A summary of these, along with methods for the other metallic elements included in this work, is presented in Table 2.

In recent years, different sorption materials have allowed the development of SPE techniques for the separation and preconcentration of Cr(III) and Cr(VI) in natural water samples, usually coupled to spectrometric techniques, achieving LODs below 1 µg L^−1^. In these, the speciation analysis was based on the different adsorption behaviors of Cr(III) and Cr(VI) on the corresponding sorbents, according to the pH range. One of these was based on a fast, simple, and selective D-µ-SPE method with magnetic graphene oxide as sorbent. Said methodology was applied to river, sea, and spring water, with detection by flame atomic absorption spectrometry (FAAS). Throughout the developed extraction procedure, strong acids and organic solvents were used, although in small quantities, limiting its environmental impact [75]. Another one of these methods was based on MSPE using magnetic NPs (MNPs) functionalized with iminodiacetic acid. This development was applied to lake and river water samples, with detection by ETAAS. In this procedure, dilute HNO_3_ was used to adjust the pH and higher concentrations of the same acid were used in the desorption stage, which generated acid residues [76]. Finally, carboxyl functionalized mesoporous silica was used for the speciation analysis of Cr by SPE coupled to ICP-MS. The developed method was applied to lake, rain, and river water samples. In this case, HNO_3_ was also used as eluent and, furthermore, the preparation of the sorbent material required the use of 100 mL of THF, which represented a significant amount of toxic waste [77]. These methods concluded that the content of Cr(III) in the studied water bodies was generally higher than that of Cr(VI), although not exceeding the legal limits, but generating alert signals due to its presence.

Rivers can become contaminated with Cr from urban, industrial, and agricultural waste that, in turn, translate into contaminated sediments. Based on this problem, a recent study was focused on the speciation analysis of this metal in sediments by means of microwave-assisted extraction, using Na_2_CO_3_ as a leaching reagent. Detection was performed by FAAS and ETAAS, and the content of Cr(III) was found to be predominant. Sample preparation required the use of a mixture of strong acids (HCl, HF and HNO_3_) to promote the digestion and subsequent determination of total Cr, which represented a significant source of hazardous waste [78]. However, HPLC-ICP-MS has been the most valuable tool for Cr speciation analysis in soil samples, due to its high sensitivity in highly complex matrices [105]. For example, a method was recently developed to determine Cr species released from soil samples collected in the contaminated area of an old tannery after an ultrasound-assisted extraction. The advantage of this method was related to the possibility of analyzing soil extracts directly, which limited the losses of Cr(VI) due to reduction processes. This approach did not report the use of appreciable amounts of strong acids and toxic organic solvents, which makes it an interesting study under the canons of Green Chemistry [79].

### 5.2. Mercury

Mercury (Hg) is one of the most hazardous elements in existence, due to its toxicity, long-distance atmospheric transport, prolonged duration in the environment, and accumulation in the food chain [106]. Hg can be found in the environment as elemental Hg, inorganic Hg(I) and Hg(II), and several organic forms, such as methylmercury (MeHg), ethylmercury (EtHg), and phenylmercury (PhHg). Due to its high volatility and susceptibility to oxidation, elemental Hg is usually present in the atmosphere, Hg(II) is the dominant species in water, soil and sediment, and MeHg is the major species in biota [107]. Among organic compounds, dimethylmercury is the most toxic species, due to its bioaccumulating capacity, extreme affinity towards macromolecules, and its slow metabolism [106].

Hyphenated techniques are the main alternatives for Hg speciation analysis, with AFS and ICP-MS being the most widely used detection techniques for its quantification. Regarding species separation, HPLC has been preferred for Hg speciation analysis due to its high separation resolution [93]. However, the direct determination of Hg species in environmental samples is usually encumbered due to its low concentrations and the complexity of many environmental matrices, making preconcentration and separation procedures usual prior to the analysis of the species of interest [106]. In this sense, SPE has been widely applied due its advantages, such as simple operation and low cost, sorbent reusability, and feasibility of online coupling [93]. In this sense, graphene oxide-bounded silica particles have been employed as SPE adsorbent for the on-line preconcentration of Hg(II), MeHg, and EtHg from river water, followed by HPLC-ICP-MS [92]. In concordance with the principles of Green Chemistry, the authors highlighted that the analysis time was very short (<5 min), minimizing the generation of toxic waste. On-line SPE with HPLC-ICP-MS for Hg speciation analysis in surface water and seawater samples has also been applied, with zwitterion-functionalized polymer microspheres as sorbent [93]. According to the results, the SPE column could be used for up to 65 extraction cycles and the Hg species were eluted in only 5 min using ammonium acetate and L-cysteine in methanol, generating minimal waste. However, large volumes of organic solvents such as acetonitrile, tetrahydrofuran, and dimethylformamide (DMF) were used to prepare and clean the polymer materials, representing a potential environmental impact.

MSPE has also been applied for Hg analysis, coupled to HPLC-ICP-MS. In this context, thioether and thiol-functionalized MNPs have been used for the preconcentration of Hg(II), MeHg, and PhHg in water and soil samples prior to their separation and quantification [94]. The results showed that 200 mL of aqueous sample could be quantitatively treated in 5 min and that the analytes could be eluted within 2 min with 0.5 mL of 0.1 mol L^−1^ HNO_3_ and 4% thiocarbamide. Hence, the method avoided the use of organic solvents and required a minimal volume of dilute acid, showing a great application potential for routine environmental sample analysis. A similar method with Fe_3_O_4_@SiO_2_@γ-mercaptopropyltrimethoxysilane MNPs as sorbent for the analysis of river and wastewater has also been reported [95]. According to the results, the HPLC separation of the three species was achieved in only 8 min but, for this, a relatively high amount of methanol—a widely known toxic solvent—had to be used in the mobile phase. Additionally, the sorbent was prepared by a chemical coprecipitation method with ethanol and NH_3_, the latter being a corrosive and volatile compound. However, this was partially superseded by the fact that the material could be reused up to 10 times.

GC-MS has also been coupled to MSPE for the preconcentration of Hg(II) and MeHg in water samples [96]. In this work, Fe_3_O_4_ NPs were modified with nanocellulose and the ionic analytes required a previous conversion into volatile derivatives. This transformation and the desorption of the resulting derivatives were achieved in a single step using 1 mL of acetic acid-acetate buffer, 0.25 mL of NaBEt_4_, and 1 mL of hexane, significantly decreasing the sample preparation time but involving the use of a volatile organic solvent.

Finally, an interesting work using a direct analyzer for the quantification and speciation analysis of Hg in sediment samples was developed [108]. Direct chemical speciation was achieved based on the different thermal behavior of the species, in a system that consisted of an electronically controlled heating unit and an atomic absorption spectrometer. The results were registered as thermodesorption curves which showed the release of the element as a function of temperature, the principle of this technique being that different species were released at different temperatures. Hence, the thermograms of the samples were compared with those of the standard Hg species for identification and quantification. An important advantage of this approach was that it did not require any sample pretreatment, except for the homogenization of the solid samples. In this way, usual digestion of solid samples with strong acids was avoided.

### 5.3. Thallium

Thallium (Tl) is a metal ubiquitously present in the environment that can generate toxic effects even at trace levels. This element is released from natural and anthropogenic sources, and exposure to it can occur via the respiratory route or through the consumption of contaminated food or water [99]. This metal is found naturally as Tl(I) and Tl(III), and both species differ in terms of bioavailability, chemical reactivity and toxicity, with Tl(III) being approximately 50,000 times more toxic than Tl(I) [100]. Tl(III) is usually found at concentrations lower than trace levels in environmental samples, therefore making its precise and accurate determination a difficult task, requiring a preconcentration step prior to its determination [100].

Recently, different approaches have been applied for the extraction and determination of Tl species. For instance, a simultaneous extraction method was performed for the determination of Tl(I), Tl(III), and total Tl in groundwater and coal mine water samples [98]. In this work, the IL Aliquat-336 and the surfactant Triton X-114 were used as ion-pair and extraction reagents of Tl(I) and Tl(III) species, which formed anionic complexes with HCl and diethylenetriaminopentaacetic acid (DTPA), respectively. The cationic and non-ionic surfactants were applied as selective extraction solvents, avoiding the consumption of oxidizing/reducing reagents and organic solvents. Moreover, the proposed approach was simple and offered a low cost, having the potential to be implemented in routine water quality monitoring. In another work, a magnetic metal-organic framework nanocomposite with MNPs was used for Tl speciation analysis in well, sea, and wastewater samples [99]. In this method, Tl(I) was selectively retained by the sorbent, and total Tl was determined using hydroxylamine hydrochloride to reduce Tl(III) to Tl(I). The authors highlighted that the method was fast, accurate and environmentally friendly, but the synthesis of the sorbent involved the use of various reagents and solvents including toluene, which is toxic, volatile, and flammable. This was partially superseded by the fact that a high sorption capacity and enrichment factor were achieved using only 20.0 mg of sorbent, and that the material could be recycled up to 15 times without affecting its performance.

Moreover, a direct separation and determination of Tl species was developed using Al_2_O_3_ functionalized with sodium dodecyl sulfate (SDS) by SPE coupled to ICP-MS, for wastewater samples analysis [100]. In this work, Tl(III) was complexed with DTPA and selectively retained and preconcentrated on the sorbent, whereas Tl(I) was not retained. The Tl(III)-DTPA complex was then eluted with HNO_3_ and detected by ICP-MS. According to the results, low LODs were obtained and, moreover, the synthesis of the self-made sorbent significantly decreased the cost of the method. However, the use of a concentrated acid as eluent was an appreciable disadvantage of this work, since it generated a partial decomposition of the sorbent, affecting its reutilization and reducing the greenness of the method. Tl speciation analysis has also been performed with microcolumns filled with 8-hydroxiquinoline immobilized onto SDS-coated Al_2_O_3_ and DTPA for selective retention of Tl(I) from soil samples, followed by ICP-MS determination [101]. In this case, the authors reported that the method was inexpensive, simple, and sensitive, although it is worth noting that large volumes of reagents were required at different stages. Moreover, Tl(I) was found to be the species with the most environmental significance in soil samples in this case.

### 5.4. Least Attended Elements

#### 5.4.1. Copper

Copper (Cu) is a metal that is widely distributed in nature, as native Cu and in its stable oxidation states, Cu(I) and Cu(II). Cu performs various functions at the cellular level, making its presence in the organism essential to ensure the proper functioning of the body, although its excess can cause damage to biomolecules. Additionally, the progressive application of Cu as an agricultural fungicide has fulfilled the homeostatic capacity of the environment, causing the tolerable concentration of this metal to be exceeded, inhibiting the growth of crops and resulting in the contamination of soils and water [109].

A method for the determination of electroactive, inert, and dissolved in acid Cu was performed at trace levels by square wave anodic stripping voltammetry in seawater samples [80]. A glassy carbon electrode coated with Au was used, which was simple, effective, and avoided pretreatment or separation steps that usually consume reagents and generate waste. In addition to this, after electrolytic cleaning, the electrode showed good measurement stability and reproducibility when reused. It is important to note the use of only small volumes of HCl (1 mL of acid per 100 mL of sample) and 0.5 mol L^−1^ H_2_SO_4_ were necessary to achieve stable and reproducible measurements. 

Recently, a method was developed for the extraction of Cu species by SPE with detection by ICP-MS in estuary, river, and sea water samples [81]. The separation of Cu(I) and Cu(II) was proposed through the use of selective complexing agents and direct extraction of Cu^0^ on an octadecyl silica cartridge used as SPE column. The sequential separation of these species also required the redissolution of residues with 2 mol L^−1^ HNO_3_, which generated a significant amount of acid waste, whereas the elution of the species required the use of ethanol. Finally, a recent report based on the analysis of the distribution of various forms of Cu in soils of different characteristics, with detection by FAAS, has also been published [82]. This analysis was performed by means of a sequential extraction procedure of six fractions. In this case, there was a predominance of specifically sorbed/bound to carbonate Cu and bound to Fe-Mn Cu. It is important to note that the proposed approach required the use of large amounts of HNO_3_ in certain stages of the sequential extraction, which implied a significant environmental impact.

#### 5.4.2. Gadolinium

Gadolinium (Gd) is a rare earth element widely used as a contrast agent in magnetic resonance imaging due to its paramagnetic properties. However, free Gd(III) generates toxic effects on human health, meaning that this species must be complexed with chelating agents for its use on patients [83]. In this respect, the removal of these Gd-based chelates is usually inefficient in wastewater-treatment plants, which results in their release to the environment, making the speciation analysis of typical Gd complexes an increasingly important task in recent years [110].

For example, six Gd-based contrast agents have been separated and determined in river water samples by hydrophilic interaction liquid chromatography coupled to ICP-MS (HILIC-ICP-MS) [83]. In this work, the use of toxic organic solvents was not required, turning this process into a green analytical method that, according to the results, gave place to low LODs for all the species. Moreover, two chelates were found in water from a wastewater treatment plant, demonstrating that the totality of these species had not been eliminated prior to the release of these waters into the environment. In another work, HILIC-ICP-MS was also applied for Gd complexes analysis in waterworks [84]. The authors indicated that Gd-DTPA was the main chelate detected in all samples and that the presence of free Gd(III) or other transformation products was not evidenced.

#### 5.4.3. Iron

Iron (Fe) is one of the most abundant elements in the Earth’s crust and it can be released into the environment from anthropogenic sources due to a wide range of applications [111]. Furthermore, Fe is an essential nutrient for living organisms, being fundamental for several cellular processes, although it can accumulate in different tissues due to its excessive intake, generating diseases such as diabetes, cirrhosis, and cancer [112]. Regarding its species, this metal is usually found in natural waters under two oxidation states: Fe(II) and Fe(III), which can be present as fractions of particulate, colloidal, and dissolved metal, as charged or neutral inorganic compounds, and as organic complexes of both species. The bioavailability of Fe depends on the chemical forms it is present as, making their determination in environmental samples highly important [85].

An approach to achieve this consisted on the application of a bulk liquid membrane containing 2-hydroxybenzaldehyde benzoylhydrazone in toluene, which allowed the selective transport of Fe(III) followed by FAAS detection in seawater samples [85]. In this work, the distribution analysis of total dissolved Fe, non-labile Fe, labile Fe(II), and labile Fe(III) fractions was performed in real seawater with good results. However, the use of toxic and volatile organic solvents such as toluene, as well as the consumption of large volumes of sample solution and reagents, can be considered a significant environmental risk. Moreover, the separation and determination of Fe species was performed on estuarine and coastal waters by on-line SPE [86]. In this work, Fe(II) was complexed with ferrozine and then extracted onto a C18 SPE cartridge, eluted with a mixture of ethanol and HNO_3_, and detected by ETAAS. For the determination of total Fe, ascorbic acid was used to reduce Fe(III) to Fe(II), thus avoiding the use of concentrated HCl and, consequently, reducing the environmental impact of the method. However, almost 7 mL of a solution composed by 30% (*v*/*v*) ethanol and 0.3 mol L^−1^ HNO_3_ was applied as eluent and 50% (*v*/*v*) ethanol was used to condition the C18 cartridges, which increased waste production. The results showed that this method was highly sensitive and accurate with low matrix interferences, which made it suitable for Fe analysis speciation in the samples under study.

#### 5.4.4. Lead

Lead and its chemical species are pollutants with high toxicity. It is widely known that organic Pb compounds are more dangerous than inorganic Pb compounds and that, among the former, tetraethyl and triethyl Pb are considered the most toxic to humans [113]. Pb enters the environment mainly through anthropogenic activities such as automobile exhaust emissions, mining, coal combustion, paint, and batteries, distributing wide amounts of this element all over the environment and ecological systems, leading to a severe hazard to the environment and to human health [87]. Regarding its speciation analysis, Yang et al. developed an on-line SPE method coupled to HPLC-ICP-MS for environmental water using graphene oxide@SiO_2_ as sorbent [87]. High enrichment factors were obtained with 10 mL of sample for trimethyl lead (TML), triethyl lead (TEL), and Pb(II) in 8 min. In this research, the use of a purely aqueous chromatographic mobile phase with sodium 1-pentanesulfonate instead of organic solvents resulted in a positive aspect regarding its environmental impact. Capillary electrophoresis coupled to ICP-MS has also been applied to determine Pb(II), TML, and TEL in algae samples, which are representative of the contamination in the water bodies from which they are obtained [88]. Experimental results showed that Pb(II), TML, and TEL were effectively separated in 20 min using an aqueous buffer solution and only 0.8000 g of dry sample, with microwave-assisted extraction. However, the numerous steps to achieve the extraction of the analytes made the proposed method cumbersome for routine analysis, in addition to the use of methanol, which is well known for its toxicity.

#### 5.4.5. Manganese

The most prevalent species of manganese (Mn) that are naturally present in water are labile Mn(II), particulate Mn(IV), and Mn(OH)_4_, and the occurrence of Mn(VII) can also be significant, mostly in relation with its use as an industrial oxidant in water treatment [90]. Of these, Mn(II) is considered an essential micronutrient, as it is necessary for the suitable functioning of the brain and of cellular metabolism, although it is well known that, at high concentrations, Mn is a toxic element that can affect the nervous system. In addition, the speciation analysis of this metal is essential because its toxicity depends on its oxidation state, following the order: Mn(II) > Mn(VII) > Mn(VI) [89].

A novel method for the speciation analysis of Mn(II) and Mn(VII) by MSPE using a nano-hybrid of a Ni–Al layered double hydroxide (LDH) and magnetite was applied to river and spring water samples [89]. The magnetic sorbent was able to selectively extract Mn(VII) through electrostatic interactions and by insertion of the negatively charged Mn(VII) species into the layers of the LDH. A fast and easy separation was achieved with the use of an external magnetic field and, in addition, the magnetic sorbent could be reused over 100 times without a significant decrease in its sorption capacity. Nevertheless, it should be noted that considerable amounts of reagents for the total Mn determination (4 mL of concentrated HNO_3_ and 2 mL of 5% *w*/*v* KIO_4_ per analysis) were used in this work. In another report, the separation and determination of Mn species was achieved in artesian water by multicommutation flow analysis-SPE [90]. Both Mn(II) and Mn(VII) ions were selectively retained by two parallel columns packed with activated silica gel and a Dowex resin, respectively. After that, large volumes of dilute HCl and of oxalic acid in HNO_3_ were applied as eluents, followed by FAAS detection. The authors highlighted that the direct determination of both ions avoided some problems related to the determination of a species by subtraction from the total analyte concentration, a calculation usually applied in previously discussed methods.

Furthermore, the speciation analysis of Mn species was performed by MSPE-ICP-optical emission spectrometry in spring, city, and lake waters [91]. In this case, Mn(VII) could be extracted with the use of an IL-β-cyclodextrin polymer combined with magnetite (Fe_3_O_4_@IL-β-CDCP) as a magnetic sorbent at a pH of 6.0, whereas total Mn was extracted at a pH of 10.0. High recovery values and enrichment factors were achieved in this method, although large amounts of reagents for the synthesis of the IL-β-CDCP were required (15 mL of DMF and 11.0 mL of hexamethylene diisocyanate in DMF and acetone).

#### 5.4.6. Tin

Organotin compounds have been used in a wide range of industrial and agricultural applications, such as biocides in agrochemicals, wood preservative fungicides, stabilizers, and catalysts for the production of poly(vinyl chloride) and antifouling paints. These compounds are extremely toxic and highly bioaccumulable, making organotin environmental pollution a severe problem. Hence, highly sensitive and selective quantification methods are necessary for its speciation analysis in environmental samples [114]. For instance, Bravo et al. developed a DLLME method for the speciation analysis of different organotin compounds in sediment samples. A mixture of methanol and tetrachloroethylene was chosen for the extraction, making the proposed method quite unfriendly to the environment. In this case, butyl-, phenyl-, and octyltin were determined by GC coupled to pulsed flame photometric detection after a 20 min extraction step [102]. A DLLME method has also been applied to extract triethyl-, tributyl-, and triphenyltin from different environmental water samples [14]. In this work, ultra-performance liquid chromatography coupled to tandem MS was used. The chromatographic elution was carried out in a gradient with a binary mobile phase, including 6 straight minutes with methanol as eluent, generating a significant amount of organic waste. Nevertheless, very low LODs were achieved and, according to the authors, the proposed method was a good alternative for the emergency monitoring of accidental environmental pollution by organotin compounds.

#### 5.4.7. Vanadium

The natural liberation of vanadium (V) into the environment mostly occurs through the weathering of dust, soil, rocks, and volcanic activities. Additionally, this element is emitted by industrial activities such as steel and iron refining, electronics and burning fossil fuels, as well as by nuclear plants. Vanadium has the dual role of essentiality and toxicity: at trace amounts it is vital for cell growth, turning toxic to plants and animals at elevated concentrations. V(IV) and V(V) species are predominant in aquatic systems, with V(V) being the most toxic [115].

A strong anion exchange SPE method for V in situ speciation analysis in groundwater samples has been reported, with ICP-MS detection [103]. In this work, the SPE cartridges were preconditioned with EDTA and V(V) was eluted by an aqueous solution of NH_4_H_2_PO_4_. Next, V(IV) was eluted using methanol, EDTA, and tetrabutylammonium hydroxide as ion-pairing reagent, the latter being a highly corrosive and environmentally toxic substance. Interestingly, this work showed that a significant conversion of V(IV) into V(V) occurred between sample collection and laboratory analysis. In another report, Tafti et al. applied a supramolecular solvent microextraction method combined with ETAAS to analyze V(IV) and V(V) in several water samples [104]. For this, a hydrophobic V(V) complex was extracted into decanoic acid reverse micelles in 400 µL of THF. Total V was then extracted after oxidation of V(IV) to V(V) with H_2_O_2_, and the concentration of V(IV) was calculated by difference. The authors emphasized that the proposed method was environmentally friendly, as the used supramolecular solvent was in good accordance with the principles of Green Chemistry.

### 5.5. Metallic Nanoparticles

Nanotechnology is, without a doubt, one of the top modern priority research areas throughout the world due to its vast potential and economic impact. This field involves the research, development, production, and processing of structures and materials on a nanometer scale in various fields of science, technology, health care, industry, and agriculture. However, the uncertainties and irregularities regarding their shape, size, and chemical composition make the presence of certain nanomaterials in the environment potentially harmful for human health [116]. Concerns have thus been raised about the destiny, transport, and transformation of released nanoparticles, which have led to the development of analytical methods designed to evaluate their presence in the environment.

In this context, silver nanoparticles (AgNPs) are recognized as one of the most frequently used nanomaterials in consumer products directly associated with human life. This has led to the inevitable discharge of AgNPs into the environment, which are known to be toxic to plants, fish, and human cells. This toxicity is believed to be linked to the release of Ag(I) ions, making the analysis of these species key to understanding their impact [117,118]. For this, a MSPE method was developed using a magnetic sorbent functionalized with poly(1-vinylimidazole) and mercaptosuccinic acid as ligand exchanger. This combination allowed the extraction and subsequent sequential desorption of Ag(I) and AgNPs with Na_2_S_2_O_3_ and HNO_3_, respectively, allowing their separate quantification by ETAAS. The method was successfully applied to lake, river, and spring water, and the sample treatment procedure involved only 0.5 mL of dilute HNO_3_, making it a fairly clean approach [119]. A CPE method was also developed for the simultaneous analysis of Ag_2_S and ZnS NPs in river, lake, and wastewater. In it, Triton X-114 was used as extractant, bis(p-sulfonatophenyl)phenylphosphane dehydrate dipotassium was used to dissolve interfering NPs, and EDTA was used to mask ionic species, with liquid chromatography coupled to ICP-MS used for separation and quantification. Overall, the method did not involve the use of harmful solvents or acids, making it a green alternative for analysis [120]. The same authors also developed a MSPE method to quantify Ag_2_S NPs and the remaining fraction of AgNPs in the same samples, by means of a treatment step with acetic acid that dissolved all AgNPs except Ag_2_S NPs [121]. The sample treatment process was devoid of any organic solvent or hazardous substance use, and the preparation of the magnetic sorbent involved only ethylene glycol as solvent, which does not have as high an impact on the environment as other organic compounds.

Au nanoparticles and Au(III) have also been simultaneously quantified. In this case, the authors developed a method for the direct analysis of samples by reversed-phase HPLC-ICP-MS, using a surfactant to improve the transport of the NPs through the column [122]. In this way, they managed to separate NPs by size during the chromatographic run although, unfortunately, the approach could not be applied to real-life samples due to the significant agglomeration of AuNPs, which hindered the separation. In turn, the speciation analysis was achieved by separation of the NPs and direct determination of Au(III), along with the treatment of a second aliquot with *aqua regia* to determine total Au, obtaining the concentration of AuNPs by difference. This method was developed without the use of major amounts of solvents and waste, except from the use of 2.5 mL of concentrated strong acids used for digestion per each 15 mL of sample.

Finally, it is worthwhile to highlight an approach for analyzing NPs of metals such as Ag, Au, and Pd, based on high-resolution–continuum source atomic absorption spectrometry. This strategy is based on the ionization delay produced by NPs —which allows their differentiation from ionic species— and on the changes in the atomization rate, which allow NPs size distinction. Nevertheless, there have been no applications of this type of methods for environmental samples to date [123].

## 6. Multielemental Speciation Analysis

So far, the discussed procedures allow single element speciation analysis. However, if the speciation analysis of several elements is required at the same time, it is necessary to propose more dynamic procedures that allow the determination of multiple species of different elements in a single analytical run, significantly reducing analysis time, lowering reagent consumption, and therefore limiting chemical waste generation. Of course, the development of this type of analysis is a demanding task, due to the need to establish optimal conditions for the separation and determination of several analytes that might present significantly different physicochemical properties. Still, without a doubt, multielemental speciation analysis is a very useful tool, particularly for the simultaneous determination of toxic elemental species in environmental samples to evaluate the degree of contamination and risk of exposure [124].

HPLC-ICP-MS is the most widely used technique in multielemental speciation analysis due to the advantages of the detector, especially concerning its ability to analyze many elements and their species in a single analytical run and to find optimal detection conditions for their simultaneous quantification. In addition, ICP-MS offers very low LODs and wide linear ranges, allowing the direct determination of analytes both at major and trace levels—as low as ng L^−1^ in some cases—without additional dilution or preconcentration of the sample [124]. Recently developed methods applying this strategy are discussed below and summarized in Table 3.

An interesting case achieved the simultaneous analysis of As(III), As(V), MMA, DMA, AB, Cr(III), Cr(VI), Sb(III), and Sb(V) in river water samples, with the aim of studying changes in the mobility and seasonality of these species in fluvial environments. The use of toxic organic solvents and strong acids was avoided throughout the whole process, which made this an interesting tool for speciation studies of these analytes in an environmentally friendly way [125]. A recent work also evaluated these species in river water and sediments. It indicated that As and Cr were found in strongly demobilized sediments, whereas Sb was mainly linked to the ion exchange fraction, thus representing a serious threat to the environment. This work concluded that As and Cr represented a medium risk whereas Sb represented a high risk in the studied samples. The authors reported the use of a mixture of strong acids and oxidants for the digestion of sediment samples, which generated acid residues that affected the overall greenness of the proposed method [126]. Finally, a similar process was developed for the speciation analysis of As(III), As(V), MMA, DMA, Cr(III), and Cr(VI) in sediment samples. It concluded that the effects of industrial pollution and the mismanagement of sewage and agricultural waste have influenced the gradual accumulation of these toxic species in the sediments under study. In this case, a digestion stage involving significant volumes of strong acids and oxidants was also proposed, although the use of organic solvents within the chromatographic separation was avoided [127].

In addition to the apparent benefits of chromatographic separations in this context, non-chromatographic multielemental speciation analysis approaches have also been reported lately. One of these was based on the use of MNPs functionalized with polyaniline as an adsorbent for the separation and enrichment of inorganic species of Se and Te. The developed MSPE-ICP-MS method was successfully applied to lake, river, and sea water samples. A selective sorption of Se(IV) and Te(IV) was achieved in the entire studied pH range, whereas the Se(VI) and Te(VI) content was calculated by difference with the total content of these analytes, obtained from a pre-reduced aliquot. As was described in their corresponding sections, this pre-reduction required the use of significant amounts of HCl, whereas the rest of the method largely avoided the use of reagents that are harmful to the environment [128]. Another approach was based on the on-line preconcentration and separation of Se(IV), Se(VI), Te(IV), and Te(VI) in samples of river, sea, and underground water, sediment and soil. The method was based on an in situ IL formation microextraction where the hydrophobic IL used as extractant was obtained on-line in a flow system. An efficient and rapid extraction of the analytes was achieved in this case, with detection by HG-AFS. Although this method required minimal reagent consumption during analysis and the use of an environmentally friendly IL was promoted, the speciation strategy was again based on a pre-reduced sample that required a significant volume of concentrated HCl [129]. In both cases, a low content of Se species was observed, and the content of Te species was below the LODs (Table 3).

## 7. Conclusions and Future Perspectives

Speciation analysis is of increasingly growing interest for analytical chemists, due to the evidence of its importance to understand the toxicity, bioavailability, and mobility of elemental species. Although this is a highly challenging task, due to the vast and diverse species an element can be present as, recent advances show that advances currently point in the right direction. In this sense, chromatographic techniques have proven to be very interesting alternatives for the speciation analysis of elements that are present as both inorganic and organic species in the environment, and also for multielemental speciation analysis, especially when coupled to high sensitivity detectors such as ICP-MS. These usually demand the use of high volumes of organic solvents as mobile phases, which generate important amounts of dangerous waste, although some of the latest advances presented in this review show the feasibility of working with modified aqueous mobile phases, reducing the environmental impact and the economic cost of analysis.

However, for cases in which only inorganic species are ecologically relevant or when only some of the species are of interest, sample treatment methods tend to be sufficient for accurate and exact speciation analysis. These represent a much more cost-effective alternative to chromatographic separations, as they can be usually coupled to more accessible detection techniques, such as AAS and AFS. Furthermore, the recent advances in the fields of nanotechnology and the implementation of modern solvent families, such as ILs, have driven sample treatment development towards a much more environmentally and economically sustainable path, thanks to the possibility of replacing traditional and hazardous organic solvents and materials by minimal amounts of these modern, greener alternatives.

This does not mean that the challenge has been completely tackled, as there is still plenty of room for environmental speciation analysis to grow and expand its reach. Fields such as the analysis of nanoparticles and of elements of growing industrial interest, such as Te, will be one of the main concerns in the near future, due to the increasing importance of these materials. In this sense, analytical advances will be followed by a deeper understanding of the importance of species-driven analysis in diverse aspects of the scientific community, which must be reflected in the development of a broader species-based legislation and environmental control. This will be aided by the development of novel techniques, materials, and methods, which should not solely aim towards achieving efficient separations and quantifications, but also towards minimizing their impact, given that it is contradictory to study the environment while damaging it at the same time. In this sense, the development of novel aqueous phases for chromatographic separations, of new solvents such as natural DESs, and of green sorbents, such as those derived from biomass, will gain a well-deserved attention, as they can open the doors to a new generation of green alternatives in environmental analytical chemistry.

## Figures and Tables

**Table 1:** Metalloid speciation analysis reports from 2016 onwards.

Species	Extraction Sorbent or Solvent	Method	Detection	LOD (µg L^−1^)	Sample/s	Ref.
Antimony						
Sb(III) and Sb(V)	Triton X-114	CPE	ETAAS	0.06	River water	[32]
Sb(III) and Sb(V)	oxMWCNTs and nano-TiO_2_	SPE	ETAAS	0.0004	River water	[33]
Sb(III) and Sb(V)	[P_6,6,6,14_][FeCl_4_]	DLLME	ETAAS	0.02	Dam, wetland, underground, rain and river water	[34]
Sb(III) and Sb(V)	Oxalic acid	Microwave extraction	HPLC-HG-AFS	0.005 and 0.008 μg g^−1^	Soils, sediments, and volcanic ashes	[35]
Sb(III) and Sb(V)	Hydroxylammonium chloride, acetic acid and EDTA, HNO_3_ and EDTA	Sequential extraction	HPL-ICP-MS	0.10 and 0.06 ng m^−3^	Particulate matter: PM2.5	[36]
Arsenic						
As(III) and As(V)	Choline chloride-phenol	DES-UALPME	ETAAS	0.01	Lake and river water, sediment and soil	[37]
As(III) and As(V)	[C_4_mim][FeCl_4_]	MIL-AALLME	ETAAS	0.03	Pond and river water, sediment and soil	[38]
As(III) and As(V)	[P_6,6,6,14_][FeCl_4_] and [P_6,6,6,14_]_3_[DyCl_6_]	MIL-DLLME	ETAAS	0.017	Dam, river, sea and underground water, sediment and soil	[39]
As(III), As(V) and phenylarsenics	Octanol	DLLME	HPLC-ICP-MS	0.001–0.039	Lake and pond water	[40]
As(III), As(V), MMA, DMA, AB and AC	2% (*v*/*v*) HNO_3_	N.A.	HPLC-ICP-MS	0.05–0.1 1–2 ng g^−1^	Fresh and salt water Sediment	[41]
As(III), As(V), MMA, DMA and roxarsone	H_3_PO_4_ and NaH_2_PO_4_ solutions	N.A.	HPLC-ICP-MS	0.24–1.52	Soil	[42]
MMA, DMA and inorganic As	Supercritical CO_2_	SFE	GC-FID	0.12–1.1 mg kg^−1^	Soils and sediments	[43]
Selenium						
Se(IV) and Se(VI)	GO-nano-TiO_2_	SPE	ETAAS	0.04	Spring water	[44]
Se(IV) and Se(VI)	Magnetic MWCNTs functionalized with Bismuthiol II	MSPE	ETAAS	0.003	River and sea water	[45]
Se(IV) and Se(VI)	Nano-SiO_2_@[C_12_mim][Br]	D-µ-SPE	ETAAS	0.0011	Rain, river, sea and underground water	[46]
Se(IV) and Se(VI)	Nano-SiO_2_	D-µ-SPE	ETAAS	0.0014	Rain, sea and underground water	[47]
Se(IV), Se(VI) and selenocyanate	Chloroform	Sequential derivatization-extraction	GC-MS	0.56, 1.67 and 0.35	Mining wastewater	[48]
Se(IV) and Se(VI)	1-undecanol	DLLME	UV-Vis	3.4	River water	[49]
Se(IV) and Se(VI)	N.A.	Hydride adsorption	UV-Vis	7 and 6	River, lake and sea water	[50]
Seven fractions	N.A.	Sequential extraction	XANES	Not reported	Agricultural soils	[51]
Six fractions	N.A.	Sequential extraction	XAS and XANES	Not reported	Phosphate mine soils	[52]
Tellurium						
Te(IV) and Te(VI)	Triton X-114 + [C_8_mim]Cl	IL-CPE	ETAAS	0.0011 and 0.0017	Sea, underground and river water, soils and sediments	[53]
Te(IV) and Te(VI)	Fe_3_O_4_@SiO_2_@PIL	MSPE	HG-AFS	0.0019 and 0.0037	Rain, underground and river water, soils and sediments	[54]
Te(IV) and CdTe NPs	N.A.	Hydride HS-SPE	ETAAS	0.13 and 0.03 (Total Te)	Superficial, lake and groundwater	[55]
Te(IV) and Te(VI)	-	-	XRF and XRAFS	Not reported	Abandoned mine soil	[56]

N.A.: does not apply, oxMWCNTs: oxidized multi-walled carbon nanotubes, UALPME: ultrasound-assisted liquid phase microextraction, AALME: air-assisted liquid phase microextraction, GC-FID: gas chromatography with flame ionization detection, GO: graphene oxide, XAS: X-ray absorption spectrometry, PIL: polymeric ionic liquid, HS-SPE: headspace solid phase extraction, XANES: X-ray absorption near edge structure, XRF: X-ray fluorescence, XRAFS: X-ray absorption fine structure.

**Table 2:** Metal speciation analysis reports from 2016 onwards.

Species	Extraction Sorbent or Solvent	Method	Detection	LOD (µg L^−1^)	Sample/s	Ref.
Chromium						
Cr(III) and Cr(VI)	Magnetized GO	D-µ-SPE	FAAS	0.10	River, sea and spring water	[75]
Cr(III) and Cr(VI)	Fe_3_O_4_@SiO_2_@IDA	MSPE	ETAAS	0.0091 and 0.0128	Lake and river water	[76]
Cr(III) and Cr(VI)	Carboxylic-functionalized nanoSiO_2_	SPE	ICP-MS	0.02	Lake, rain and river water	[77]
Cr(III) and Cr(VI)	Na_2_CO_3_	MAE	FAAS and ETAAS	0.02 and 0.03 µg g^−1^	Sediment	[78]
Cr(III) and Cr(VI)	Na_2_CO_3_	UAE	HPLC-ICP-MS	0.08 and 0.09	Soil	[79]
Copper						
Electroactive, inert and acid-dissolved Cu	Au/Glassy carbon electrode	N.A.	SWASV	0.5–1.1 nmol L^−1^	Seawater	[80]
Cu^0^, Cu(I) and Cu(II)	Octadecyl silica	SPE	ICP-MS	0.8 ng kg^−1^	Estuarine, river and sea water	[81]
Cu fractions	NH_4_OAc and NH_2_OH·HCl	Sequential extraction	FAAS	N.R.	Soil	[82]
Gadolinium						
Six Gd complexes	N.A.	N.A.	HILIC-ICP-MS	0.0034–0.022	River water	[83]
Three Gd complexes	N.A.	N.A.	HILIC-ICP-MS	8.11–14 pmol L^−1^	Waterworks	[84]
Iron						
Fe(II) and Fe(III)	2-hydroxybenzaldeyde benzoylhydrazone in toluene	Liquid membrane extraction	FAAS	N.R.	Sea water	[85]
Fe(II) and Fe(III)	C18 SPE cartridge	SPE	ETAAS	1.38 nmol L^−1^	Estuarine and coastal waters	[86]
Lead						
Pb(II), TML and TEL	GO@SiO_2_	SPE	HPLC-ICP-MS	0.000018 (TML) and 0.000023 (TEL)	River water	[87]
Pb(II), TML and TEL	N.A.	N.A.	CE-ICP-MS	0.091, 0.023 and 0.030	Algae	[88]
Manganese						
Mn(II) and Mn(VII)	Ni–Al LDH/Fe_3_O_4_	MSPE	FAAS	0.1	River and spring water	[89]
Mn(II) and Mn(VII)	Activated silica gel, Dowex resin	SPE	FAAS	1.4 and 4.8	Artesian water	[90]
Mn(II) and Mn(VII)	Fe_3_O_4_@ILs-β-CDCP	MSPE	ICP-OES	0.15 and 0.27	Spring water, city water and lake water	[91]
Mercury						
Hg(II), MeHg and EtHg	GO@SiO_2_	SPE	HPLC-ICP-MS	0.005, 0.006 and 0.009	River water	[92]
Hg(II), MeHg and EtHg	Zwitterion-functionalized polymer microspheres	SPE	HPLC-ICP-MS	0.78, 0.63 and 0.49	Surface and sea water	[93]
Hg(II), MeHg and PhHg	Fe_3_O_4_@SiO_2_@GMA-S-SH	MSPE	HPLC-ICP-MS	0.40, 0.49 and 1.4	Farmland water and soil	[94]
Hg(II), MeHg and PhHg	Fe_3_O_4_@SiO_2_@γ-MPTS	MSPE	HPLC-ICP-MS	0.74, 0.67 and 0.49	River and wastewater	[95]
Hg(II) and MeHg	Fe_3_O_4_ modified with nanocellulose	MSPE	GC-AFS	5.6 and 4.0	River water	[96]
Hg(II) and MeHg	Triton X-114	CPE	HG-AFS	7 and 18	Industrial wastewater	[97]
Thallium						
Tl(I) and Tl(III)	Aliquat-336 and Triton X-114	LL-MM-CPE	ETAAS	0.015	Groundwater and coal mine water	[98]
Tl(I) and Tl(III)	Fe_3_O_4_@SiO_2_@ADB18C6 and MIL-101(Cr)	MSPE	ETAAS	0.0015	Well and sea water and wastewater	[99]
Tl(I) and Tl(III)	Al_2_O_3_ functionalized with SDS	SPE	ICP-MS	0.025 and 0.16	Wastewater	[100]
Tl(I) and Tl(III)	Oxine immobilized in SDS-coated Al_2_O_3_	SPE	ICP-MS	0.037 and 0.18	Soils	[101]
Tin						
Butyl-, phenyl- and octyltin	Tetrachloroethylene	DLLME	GC-PFPD	0.3–1	Sediments	[102]
TET, TBT, TPhT	Chlorobenzene	DLLME	UPLC-MS/MS	0.003–0.010	Sea, river and lake water	[14]
Vanadium						
V(IV) and V(V)	SAX cartridge	SPE	ICP-MS	0.05	Groundwater	[103]
V(IV) and V(V)	Decanoic acid in THF	SSME	ETAAS	0.0012	River and seawater	[104]

N.A: does not apply, N.R.: not reported, GO: graphene oxide, IDA: iminodiacetic acid, SWASV: square-wave anodic stripping voltammetry, CE: capillary electrophoresis, GMA: glycidyl methacrylate, γ-MPTS: γ-methacryloxypropyl trimethoxysilane, LL-MM-CPE: ligandless mixed micelle cloud point extraction, ADB18C6: aminodibenzo-18-crown-6, PFPD: pulsed flame photometer detection, SAX: strong anion exchange, SSME: supramolecular solvent microextraction.

**Table 3:** Multielemental speciation analysis reports from 2016 onwards.

Species	Extraction Sorbent or Solvent	Method	Detection	LOD (µg L^−1^)	Sample/s	Ref.
Multielemental speciation analysis						
As(III), As(V), MMA, DMA, AB, Cr(III), Cr(VI), Sb(III) and Sb(V)	Na_2_HPO_4_, KH_2_PO_4_ and Na_2_EDTA	-	HPLC-ICP-MS	0.009–0.37	River water	[125]
As(III), As(V), MMA, DMA, AB, Cr(III), Cr(VI), Sb(III) and Sb(V)	pH buffers and Na_2_EDTA	-	HPLC-ICP-MS	0.013–0.096	River water and sediment	[126]
As(III), As(V), MMA, DMA, Cr(III) and Cr(VI)	(NH_4_)_2_HPO_4_ and EDTA	-	HPLC-ICP-MS	0.064–0.682 ng g^−1^	Sediment	[127]
Se(IV), Se(VI), Te(IV) and Te(VI)	Fe_3_O_4_@SiO_2_@polyaniline	MSPE	ICP-MS	1.2–5.3 ng L^−1^	Lake, river and sea water	[128]
Se(IV), Se(VI), Te(IV) and Te(VI)	[C_8_mim]PF_6_	IL-ISFME	FI-HG-AFS	1.8–3.2 ng L^−1^	River, sea and underground water, sediment and soil	[129]

IL-ISFME: In situ ionic liquid formation microextraction.
